# Direct-Dispense Polymeric Waveguides Platform for Optical Chemical Sensors

**DOI:** 10.3390/s8127636

**Published:** 2008-12-01

**Authors:** Mohamad Hajj-Hassan, Timothy Gonzalez, Ebrahim Ghafar-Zadeh, Hagop Djeghelian, Vamsy Chodavarapu, Mark Andrews, Daniel Therriault

**Affiliations:** 1 Department of Electrical and Computer Engineering, McGill University, 3480 University Street, Montreal, Quebec, Canada H3A 2A7; E-Mails: mohamad.hajjhassan@mail.mcgill.ca (M. H. H); Ebrahim.ghafarzadeh@mail.mcgill.ca (E. G. Z.); 2 Department of Chemistry, McGill University, 801 Sherbrooke Street West, Montreal, Quebec, Canada H3A 2K6; E-Mails: timothy.gonzalez@mail.mcgill.ca (T. G.); hagop.djeghelian@mail.mcgill.ca (H. D.); mark.andrews@mcgill.ca (M. A.);; 3 Department of Mechanical Engineering, Ecole Polytechnique de Montreal, Case postale 6079, Succursale Centre-ville, Quebec, Canada H3C 3A7; E-Mail: daniel.therriault@polymtl.ca (D. T.)

**Keywords:** Direct-Dispense, Direct-Write, Xerogels, Oxygen Sensors, Waveguides, Optical Sensors, Fluorescence, Chemical Sensors, Polymer Waveguides

## Abstract

We describe an automated robotic technique called direct-dispense to fabricate a polymeric platform that supports optical sensor arrays. Direct-dispense, which is a type of the emerging direct-write microfabrication techniques, uses fugitive organic inks in combination with cross-linkable polymers to create microfluidic channels and other microstructures. Specifically, we describe an application of direct-dispensing to develop optical biochemical sensors by fabricating planar ridge waveguides that support sol-gel-derived xerogel-based thin films. The xerogel-based sensor materials act as host media to house luminophore biochemical recognition elements. As a prototype implementation, we demonstrate gaseous oxygen (O_2_) responsive optical sensors that operate on the basis of monitoring luminescence intensity signals. The optical sensor employs a Light Emitting Diode (LED) excitation source and a standard silicon photodiode as the detector. The sensor operates over the full scale (0%-100%) of O_2_ concentrations with a response time of less than 1 second. This work has implications for the development of miniaturized multi-sensor platforms that can be cost-effectively and reliably mass-produced.

## 1. Introduction

Multi-sensor systems and sensor microarrays are undergoing rapid development because they can multiplex and de-multiplex biological or chemical information from multi-component samples [1, 2]. Optical sensing based on fluorescence spectroscopy is a well established technique for highly selective and sensitive biochemical sensing [3, 4]. In comparison to competing approaches, optical sensors may be less susceptible to contamination while providing fast multiplexed response and high efficiency. Optical sensor arrays have proven attractive for DNA-chip technology and protein microarrays widely being used for drug discovery, genomics and proteomics [5, 6]. Biochemical sensor arrays are also seeing development in applications like point-of-care diagnostics, medical/clinical biosensing, personal/food safety, and environmental monitoring [3, 7]. To date, a number of platforms have been demonstrated for optical biochemical sensor arrays. Some platforms use optical fibers [8], planar waveguides [9], some of which have been coupled with micro-LED arrays [10], and arrays on a LED [11].

We work on waveguide based sensors which offer a degree of robustness and that can be integrated relatively easily into microfluidic structures for lab-on-chip applications [12]. The waveguide structures act as a support platform for immobilizing biochemical recognition elements. We describe a direct-dispense fabrication process to implement the waveguide based sensor arrays. Direct-dispense and direct-write techniques are commonly used for advanced microelectronic packaging, microfabrication, and microelectrodes development [13, 14]. With direct-dispense, select materials can be precisely deposited with features on the order of several micrometers through a microscale nozzle. A longer term objective is to evaluate the potential of direct-dispense techniques for low-cost printable optical sensor arrays. Recently, a number of research groups including ours have employed the direct-dispense technique to create microfluidic channels and other microstructures [15, 16]. In literature, we find several fabrication techniques that have been grouped as direct-write techniques which include laser (optical) lithography, ink-jet printing and screen printing [17]. The fabrication technique described here is one example of the several direct-write techniques and involves mechanical robotic deposition to pattern fugitive organic inks on a substrate. Optically transparent epoxy or polymeric materials are then deposited with direct-dispense system using the previously deposited fugitive ink structures as guide or sacrificial layers in a way that is reminiscent of the use of positive photoresists. Subsequently, the fugitive ink is thermally removed leaving behind micropatterned epoxy or polymeric structures. This process can be repeated to create complex polymeric microstructures that can be used as high-performance, multi-functional sensor platforms which can support microfluidics and optical signal collection and transmission.

**Figure 1.**
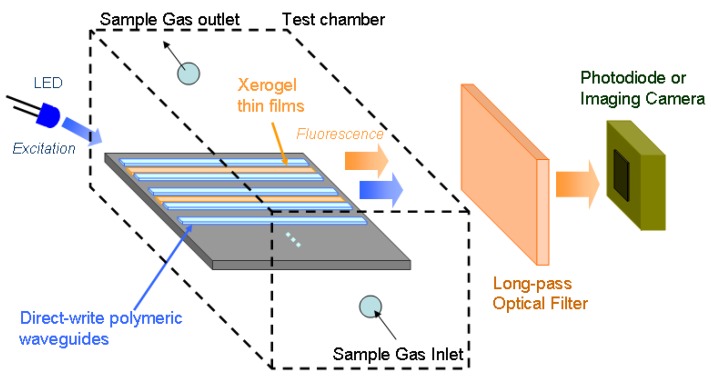
Simplified block diagram of the optical sensor system implementing the polymeric waveguide support platform and xerogel recognition materials.

Sol-gel derived materials have a good potential for creating sensor arrays in combination with direct-dispense techniques. Several reports describe the use of sol-gel-processed xerogels as encapsulation media for various biochemical recognition elements [7, 18, 19]. Xerogels offer a number of advantages, including low temperature processing, optical transparency, relative long time stability, and straightforward doping procedures to encapsulate guest sensing elements. Xerogel sensor materials have been deposited using pin-printing [7], dip-coating [8], spin-coating [20], and photo-(ultraviolet) patterning [21]. This flexibility makes xerogels attractive to combine with the direct-dispense method. In this paper, we show how modified xerogels can be deposited selectively and integrated with a patterned polymeric support platform using direct-dispensing. Ru(II) diimine complexes have relatively long lived triplet metal-to-ligand charge transfer states that make them useful for quenchometric sensing. We use *tris*(2,2′-bipyridyl)dichlororuthenium(II) hexahydrate ([Ru(bpy)_3_]^2+^·6H_2_O) as the guest luminophore in the xerogel, because it is one of the more widely studied complexes for oxygen sensing by luminescence quenching. For the prototype discussed in this paper, we use an optically transparent epoxy Epo-Tek 301-1 (Epoxy Technologies Inc.) to fabricate polymer ridge waveguides. Figure 1 shows a simplified block diagram of the prototype optical sensor platform implementing the polymeric waveguide support platform and xerogel sensor materials. Other configurations common to existing optical chemical sensors are also possible where the excitation source and the photodetector are on the either side of the xerogel sensor array or both the excitation source and the photodetector are on the same side (top or bottom) of the sensor array [12, 22-24].

Following Figure 1, the sensor partly uses direct LED excitation and partly guided evanescent-wave excitation due to the exponentially decaying evanescent field from the multimode waveguides [8, 25]. Subsequently, fluorescence is generated from Ru(II) complexes trapped in the xerogel claddings with an intensity proportional to the target analyte concentration. A part of this generated fluorescence is captured by the waveguides and channeled to a photodetector. In the following sections, we describe the direct-dispense technique to create the waveguide structures, preparation of the modified xerogel sensor materials that are suitable for direct-dispense process, and finally, the integration of a xerogel sensor film with the waveguide structures.

## 2. Direct-dispense fabrication of Waveguide structures

**Figure 2.**
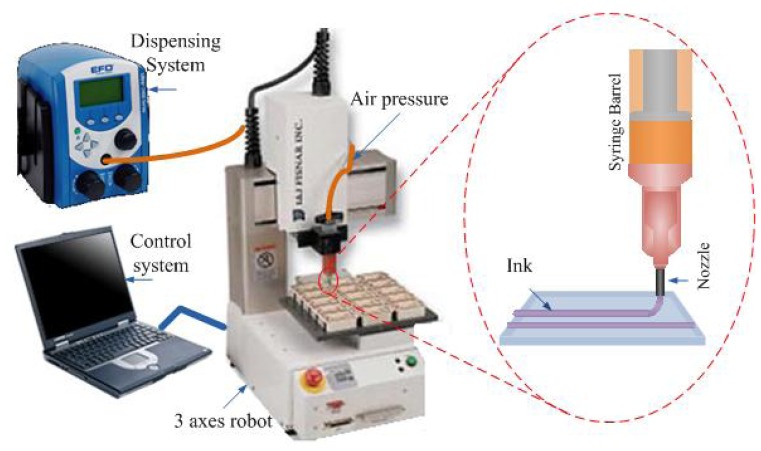
Direct-dispense microfabrication system.

**Figure 3.**
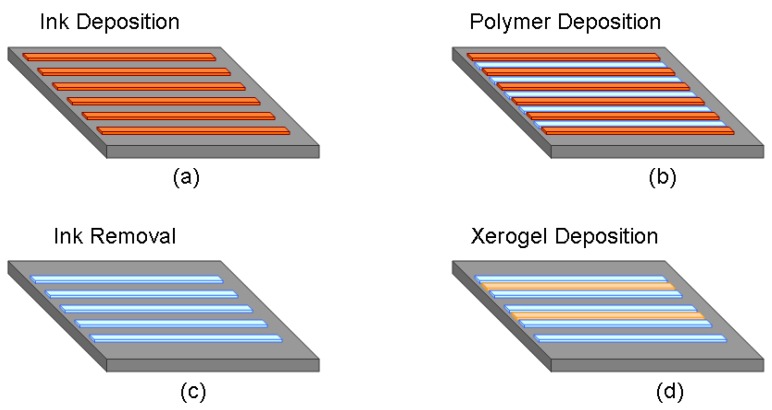
Fabrication process flow for the development of polymeric waveguide support platform using direct-dispense process and the immobilization of xerogel mateirals.

**Figure 4.**
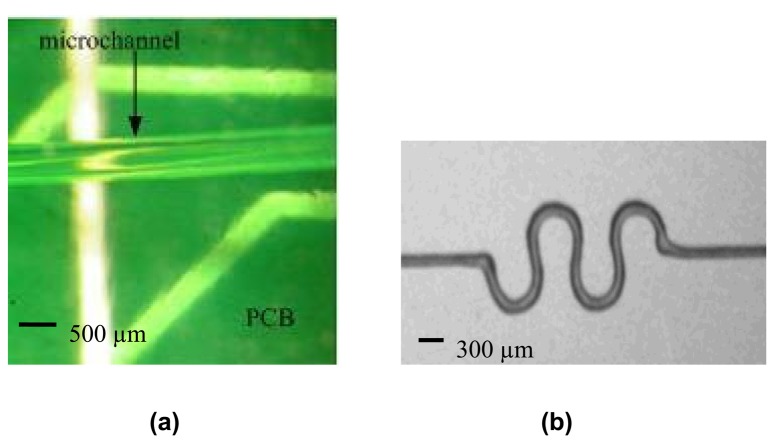
Microstructures fabricated using direct-dispense technique. (a) Microscopic image of a fabricated 500 μm diameter microchannel on a Printed Circuit Board (PCB). (b) Serpentine microchannel with an inner diameter of 150 μm and 200 μm outer diameter.

A robotic deposition tool (Model I&J 2200, FISNAR Inc.) was used for the direct-dispense process, in which a fugitive ink, typically consisting of a composite of 75% of petroleum jelly and 25% of microcrystalline wax, was extruded through a micro-nozzle (Model 2400, EFD Inc.) and deposited onto a substrate. Typical substrates may be selected from plastic, generic microscope borosilicate glass slides, or fragments of silicon wafers [15, 16]. The automated direct-dispense tool is as shown in Figure 2. The pattern to be written is first mapped out as a trajectory by programming it into the robot, which in turn commands and controls the dispensing system to emit the fugitive ink from the nozzle. In the simplest case (Figure 3), straight lines of the fugitive ink are deposited. Next, uncured liquid polymer resin is deposited at room temperature with the direct-dispense apparatus. The fugitive ink pattern acts as a periodic reservoir to hold the fluid resin, which is subsequently cured. The ink is then melted at 65°C and extracted, leaving behind patterned polymeric microstructures. Figures 3 (a), (b), and (c) depict the fabrication steps for the direct-dispense process. The fugitive ink layer must have appropriate flow viscosity, solidify and adhere well to the substrate and melt at a sufficiently low temperature to yield uniform quality and reproducible structures from polymer resins. As an example, Figure 4 shows functionality of the direct-dispense system to fabricate different microchannels.

**Figure 5.**
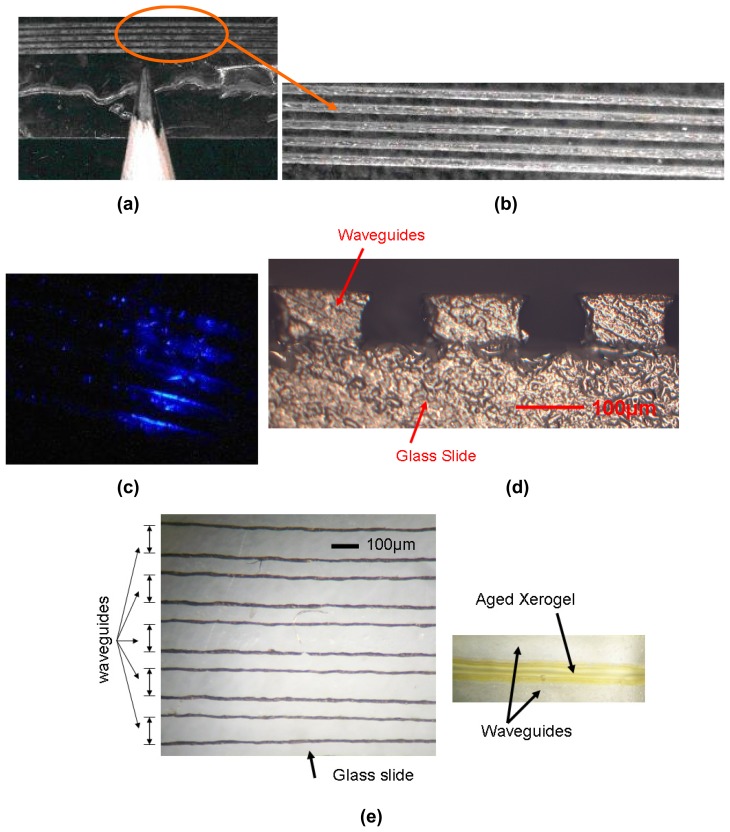
Fabricated epoxy waveguides using the direct-dispense deposition technique. (a) Shows the top-view of the fabricated 100 μm wide ridge waveguides spaced 75 μm apart with a pencil tip as reference; (b) Magnified top-view of a selected portion of the waveguides; (c) Multimode epoxy waveguides which conduct light from a blue (λ_peak_ = 470 nm) LED; (d) Cross-sectional view of the waveguides; (e) Magnified top-view of the transparent waveguides under intense optical illumination which shows the straightness and reproducibility of the structures. Figure (e) also shows the deposited xerogel after aging.

Accordingly, the direct-dispense system is programmed to make multimode ridge waveguides 100 μm in width, 100 μm thick and several millimeters in length. First, 100 μm lines of ink spaced 75 μm apart were deposited on a borosilicate glass slide. A layer of low viscosity, optically transparent epoxy Epo-Tek 301-1 was then deposited in the periodic reservoirs between the lines to form the incipient waveguides. The Epo-Tek 301-1 polymerized after standing and curing at room temperature for 24 hours. Cured Epo-Tek 301-1 adheres tenaciously to a wide range of substrates and exhibits low shrinkage, so that the ink effectively acts as a mold. Epo-Tek 301-1 has an optical transparency of >96% between 400 nm and 1,600 nm wavelengths. The last step in the fabrication of the epoxy waveguides is the removal of ink lines located between the epoxy guides. The stripping step was performed by placing samples in an oven or on a hot plate maintained at 65°C. In effect, the fugitive ink is a positive resist. Figure 5(a) shows the top-view of the fabricated 100 μm wide ridge waveguides spaced 75 μm apart with a pencil tip as reference. Figure 5(b) shows a magnified top-view of a selected portion of the waveguides. Figure 5(c) shows multimode epoxy waveguides which conduct light from a blue (λ_peak_ = 470 nm) LED. Figure 5(d) shows cross-sectional view of the waveguides. In Figure 5(d) we notice a semi-circular structure to the side-walls for the waveguides. This structure is due to the cylindrical structure of the fugitive-ink lines. Figure 5(e) shows a magnified top-view of the waveguides that are transparent under intense optical illumination. This image will help in understanding the straightness and fabrication reproducibility of the waveguides. Figure 5(e) also shows the deposited xerogel between waveguides after aging for 5 days. Typically, the microstructures produced using the proposed direct-dispense technique are about 5-10% inaccurate depending on the dimensions of the targeted structures. This inaccuracy increases as the dimensions of the structures reduce to the system limit of 10 μm. The structural variations are mainly due to ambient vibrations that affect the nano-manipulation system controlling the robotic deposition arm of the direct-dispense system.

## 3. Xerogels based biochemical sensor arrays

In addition to the waveguide element for delivery and receiving optical signals, the xerogel sensing film is an important element of the array. Xerogels are micro-/nano-structured porous glasses that have been extensively researched for the development of sensors for various biochemical analytes including oxygen, glucose, pH, and lactate [7, 10, 19, 26]. The appeal of xerogels for biochemical recognition elements derives from their thermal stability, tunable pore dimensions and distributions, adjustable pH and dipolarity, a broad optical transparency window, and evidence that they can be used to sequester a wide variety of active agents like luminophores in their porous networks. In the area of xerogel based biochemical sensor arrays, Cho *et al.* [7] have explored pin-printing to create xerogel sensor arrays. Aubonnet *et al.* [21] introduced photopatterned waveguides for sensing, but the performance of the sensors was limited to the detection of gaseous samples because waveguide transmission losses were too large in sol-gel samples that readily hydrated. More recently, Burke *et al.* [22, 27] developed embossing techniques to create sol-gel waveguides. They used pin-printing to integrate xerogel materials with the waveguides. The direct-dispense microfabrication technique described here is comparable to sol-gel processing techniques for the waveguides development in terms of ease of fabrication and potential low cost. The use of direct-dispense technique for the waveguide microstructures can further improve the integration and functionality of sensor microsystems by combining the properties of gaseous/fluidic sample manipulation and sensing.

Real-time monitoring of O_2_ is important for blood gas analysis, water treatment, and food quality analysis. More than often, optical oxygen sensor systems are based on the measurement of luminescence intensity. Here, we prototype an O_2_ sensor developed from the fugitive ink direct-dispense process. The detection scheme invokes fluorescence quenching of physically doped triplet [Ru(bpy)_3_]^2+^ [3, 28], whose quenching by oxygen has been quite well-studied [7, 10, 26]. Luminophore quenching in xerogel media is governed by many factors, and in the simplest scenario of a homogeneous distribution of luminophore molecules with a single exponential decay, luminescence quenching is described by the standard Stern-Volmer expression of Equation (1). In this expression, *I_0_* and *τ_0_* are the luminescence intensity and lifetime in the absence of analyte respectively, *I* is the luminescence intensity in the presence of analyte, *K_SV_* is the Stern-Volmer constant, *k_q_* is the bimolecular quenching constant and A is the fractional analyte concentration [28].

(1) $$\frac{I_{0}}{I}=1+K_{SV}\;[A]=1+K_{q}\tau_{0}\;[A]$$

It is recognized that physical doping and even covalent binding of the luminescent probe can give rise to heterogeneities in the local environment of the guest to cause nonlinear SV plots [29]. Two-site models, systematic changes in the host xerogel composition and more complex covalent attachment schemes have all been used to linearize and improve the SV response [30]. These strategies, desirable as they are, lie outside the scope of our current work which is not that of sensor optimization; instead our focus is to demonstrate functionality of a printable sensor array created by the fugitive ink direct-dispense technique.

### 3.1. Preparation of the Xerogel Oxygen (O_2_) Sensors

We prepared what is commonly referred to as a Class II hybrid xerogel. This material abandons the classic combinations of tetraethoxyorthosilicates (TEOS) and tetramethoxyorthosilicates (TMOS) in favor of a silicon alkoxide component that is organically modified with a nonhydrolyzable covalent C-Si bond. Moreover, and anticipating future studies, the organic substituent can be a latently reactive moiety like a polymerizable monomer that can be further used to alter the properties of the host matrix (through crosslink density). Thus, the *sol* was prepared by acid catalysed hydrolysis and polycondensation of 3-methacryloxypropyltrimethoxysilane (MAPTMS) in combination with methacrylic acid and ZrO_2_. Accordingly, MAPTMS (10.14 g, 0.040 mol) was combined with 0.05M hydrochloric acid (HCl, 0.54 g). The solution was stirred and aged for 1 hour at room temperature. During this time, a separate solution was prepared from 70% zirconium(IV)propoxide (6 g) in 1-propanol (0.013 mol) and methacrylic acid (1.1 g, 0.013 mol). These were combined and aged for 40 minutes. The methacrylic acid is incorporated to chelate Zr^4+^ in order to suppress the otherwise rapid hydrolysis that would deposit large particles of light scattering zirconia when the zirconium alkoxide is combined with the aqueous MAPTMS. After 1 hour, the MAPTMS solution is added drop-wise to the zirconium solution. The resulting *sol* is further hydrolyzed by the addition of distilled water (0.98 g) and stirred while being aged for at least 16 hours. The final ratio of water to silicon (r-value) is 1.5. The fluorescence sensor was prepared from a 1:4 mixture of 10 mmol/L *tris*(2,2′-bipyridyl) dichlororuthenium(II) hexahydrate in water and MAPTMS *sol*. 400 μL aliquots of [Ru(bpy)_3_]^2+^·6H_2_O were added to MAPTMS *sol* (1,400 μL). Rapid *gel* formation was avoided by adding methanol (1 mL) containing water (200 μL) to the final *sol*.

Prior to xerogel deposition, the waveguide samples were diced using a semiconductor-dicer to a length of 1 centimeter. The exposed glass substrate and epoxy waveguides were cleaned with isopropanol. The epoxy surface was first coated with the adhesion promoter, 3-aminopropyltriethoxy-silane (APTS). The APTS was dried at 50°C for 2 hours. Once dry, the epoxy surface was again cleaned with isopropanol. Sol-gel sensing layer was subsequently deposited by direct-dispense in the spaces left by the stripped ink between two epoxy waveguide structures. The resulting structure of the sensor is shown in Figure 3(d). The hybrid glass was then dried for 1 hour at 50°C before a second layer was applied using the same procedure. The sensor waveguide structures were dried at 50°C for five days.

## 4. Experimental Results and Discussion

The experimental setup for testing the proposed planar ridge waveguides is similar to the system shown in Figure 1. The setup consists of a blue LED (Digikey, λ_peak_: 470 nm) for exciting the xerogel coated waveguide structures on the glass slide. The LED is sinusoidally modulated at 400 Hz with peak-peak sinusoidal amplitude of 1 V and DC offset of 2.7 V. The resulting optical signal collected by the epoxy waveguides is passed through a long pass (Thorlabs: FGL590, λ_cut-off_: 590 nm) optical filter to remove the excitation light and allow only the fluorescence emission to pass through. The fluorescence signal is detected by a standard silicon photodiode (Thorlabs: PDA100A) and the output of the photodiode is recorded by a lock-in amplifier (Stanford Research Systems, SRS830). Both the excitation LED and photodetector are placed as close as possible (less than 5 mm) to the waveguide structures to maximize the efficiency in the sensing process. The sensor response is measured as a function of gaseous O_2_ concentration at room temperature (25°C). The O_2_ concentration is varied by changing its relative percentage with respect to gaseous N_2_ concentration. The gases are mixed within a custom built gas mixing manifold, consisting of a matched pair of flow controllers (Cole-Parmer: EW-03229) connected to O_2_ and N_2_ gas cylinders.

As a prototype using the direct-dispense process, we fabricated a linear array of five polymer waveguides which housed two xerogel waveguides. As explained earlier, this configuration was used mainly for two reasons (i) xerogels are in aqueous form and hence two waveguide structures are used to contain the solution between them during deposition and (ii) the waveguide spacing between consecutive sensors is maintained in order to clearly distinguish the response from each sensor in the array. The sensor platform is scalable and can be used to implement large scale arrays for multi-sensor systems. As introduced earlier, the sensors operate on the basis of evanescent wave sensing [8, 25]. In the prototype system we used a photodiode as the detector which can only measure the cumulative fluorescence response from the sensor array. Alternatively, using a Charge Coupled Device (CCD) camera as the detector would enable us to distinguish the response from each sensor in the array.

**Figure 6.**
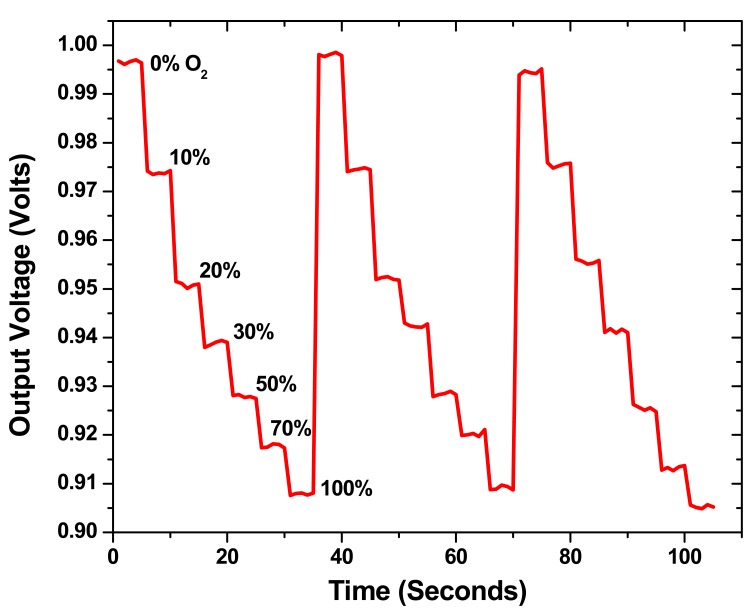
Response of the O_2_ responsive xerogel sensor materials.

**Figure 7.**
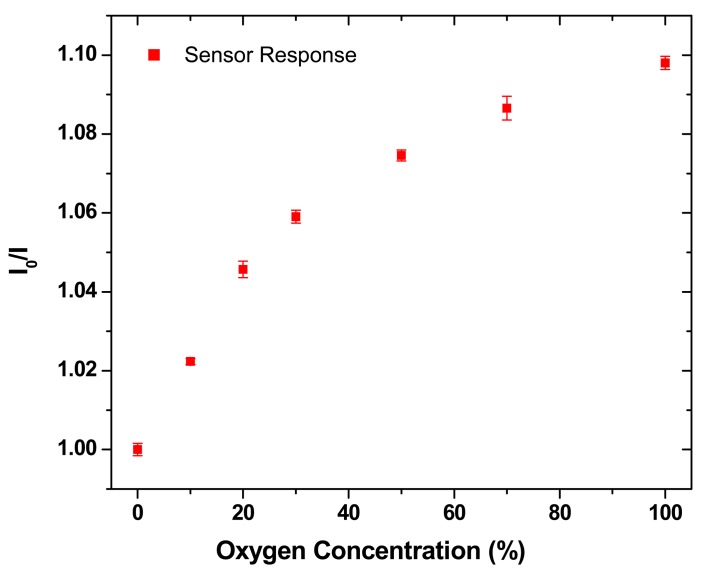
Stern-Volmer response of the xerogel O_2_ sensors.

Figure 6 shows the response of the sensor as continually recorded by the lock-in-amplifier from the photodiode output by varying the oxygen concentration with respect to nitrogen gas. From the graph, we notice that the sensor system has repeatable performance with a response time on the order of 1 second. Measuring the exact response of the xerogel sensor material is difficult and the measured time is the experimental system response time which includes the time taken by the gas concentration to flow from the tanks through the controllers and tubing and fill the test chamber. Figure 7 shows the Stern-Volmer plot for the xerogel sensors. Each datum is the mean of results from multiple measurements. While the Stern-Volmer plot is nonlinear [28] for reasons discussed previously in Section 3, the data in Figures 6 and 7 show that the basic concept of the sensor array has been demonstrated. We notice that the sensitivity of the sensor film is comparable to optical sensors that are based on printable technologies developed by other research groups [21, 22, 27]. However, the sensitivity is lower than sol-gel sensor materials that specifically aim for increased sensitivity [18, 30]. The xerogel formulation has been modified from traditional recipes (which are typically immobilized with dip coating or spin coating) to avoid rapid *gel* formation from *sol* state. This modification is a novel step that renders the xerogel immobilization compatible with direct-dispense fabrication process. We are currently working towards improving the sensitivity of the xerogel materials that can be immobilized with the direct- dispense technique.

## 5. Conclusions

We have described the implementation of direct-dispense patterning to develop polymeric waveguide support platform for optical multi-sensor systems. Specifically, we demonstrated a waveguide array structure made with direct-dispense to support sol-gel derived xerogel recognition materials which are made responsive to oxygen concentration. The direct-dispense fabrication process suggests a scalable, versatile, cost-effective technology towards printable optical sensor arrays. The direct-dispense process may in the future lead to a high-performance, multi-functional sensor platform that can support gaseous/fluidic sample handling and optical signal collection and transmission. Advantages of this technique as compared to other soft-microfabrication techniques, are that one needs only a single direct-dispense setup (no requirement for combining multiple fabrication strategies or the creation of micro-contact printing masters), and ability to develop structures on–the-fly. The developed xerogel sensors performed for the full-scale range (0%-100%) of gaseous oxygen concentration with a response time of less than 1 second. The sensors show comparable sensitivity to other competent optical chemical sensors based on printable and patternable technologies. However, the sensitivity needs to be improved for practical field-usable applications. This is not unexpected, as more research in general is required to develop high sensitivity xerogel sensor materials. Nonetheless, the findings suggest a bright outlook for miniaturized multi-sensor platforms by direct-dispense.
